# Dynamic change of soluble interleukin-2 receptor distinguished molecular heterogeneity and microenvironment alterations in diffuse large B-cell lymphoma

**DOI:** 10.1186/s40364-022-00401-4

**Published:** 2022-07-25

**Authors:** Yu-Jia Huo, Peng-Peng Xu, Li Wang, Hui-Juan Zhong, Di Fu, Qing Shi, Shu Cheng, Shuo Wang, Mu-Chen Zhang, Wei-Li Zhao

**Affiliations:** 1 Shanghai Institute of Hematology, State Key Laboratory of Medical Genomics, National Research Center for Translational Medicine at Shanghai, Ruijin Hospital Affiliated to Shanghai Jiao Tong University School of Medicine, 197 Rui Jin Er Road, Shanghai, 200025 China; 2 Pôle de Recherches Sino-Français en Science du Vivant et Génomique, Laboratory of Molecular Pathology, Shanghai, China

**Keywords:** Diffuse large B-cell lymphoma, sIL-2R, Dynamic change, Prognosis, Lymphoma microenvironment

## Abstract

Diffuse large B-cell lymphoma (DLBCL) is an aggressive lymphoma with variable clinical outcomes and prediction of prognosis remains important for long-term remission. We performed serial serum soluble interleukin-2 receptor (sIL-2R) measurement pretreatment and before each cycle of the treatment in 599 patients with de novo DLBCL. Genomic and transcriptomic features were analyzed by 223 DNA- and 227 RNA-sequencing, respectively. Applying the cut-off value to sIL-2R pretreatment and cycle 2 (C2) level, patients were classified into FINE subtype (pretreatment low level) with good prognosis, RES subtype (pretreatment high level and C2 low level) with intermediate prognosis, and RET subtype (pretreatment high level and C2 high level) with poor prognosis, independent of International Prognostic Index. In “others” genetic subtype, dynamic change of sIL-2R showed prognostic significance and genetic features. Compared with FINE subtype, RES subtype had increased *ARID1A* and *MYD88* mutations, and RET subtype had increased *KMT2D*, *LYN* and *SOCS1* mutations. RES and RET subtypes showed significant enrichment in oncogenic pathways, such as ERK, NF-κB, JAK-STAT, and immune-associated pathways. As for tumor microenvironment, RES subtype exhibited increased recruiting activity of CD8 + T, T helper 1, and natural killer cells, and RET subtype with increased recruiting activity of CD4 + T and regulatory T cells in silico. There was a positive correlation between transcripts of IL-2R and immune checkpoint expressions including PD-1 and CTLA-4. Our findings identified that dynamic change of sIL-2R, with this simple and easy detection method in peripheral blood, had long-term prognostic effect and specific relation to microenvironment alterations in DLBCL.

**Supplementary Information:**

The online version contains supplementary material available at 10.1186/s40364-022-00401-4.

To the Editor,

Diffuse large B-cell lymphoma (DLBCL) is the most common subtype of lymphoma with clinical and molecular heterogeneity [1]. Due to the limitations in tumor sample availability, simple and easy detection methods in peripheral blood are important for surveillance of clinical response [2–5]. The interleukin (IL)-2 and IL-2 receptor (IL-2R) interplay regulates immune response by activating multiple signaling pathways, including Janus kinase (JAK)-STAT, nuclear factor (NF)-κB, and extracellular signal-regulated kinase (ERK) pathways [6]. Soluble IL-2R is produced by immune cells, including regulatory T (Treg) cells [7]. Here we performed serial serum measurement of sIL-2R in 599 patients with de novo DLBCL (Fig. S1a). Median sIL-2R level pretreatment and before cycle 2 (C2) to cycle 6 were 697, 527, 564, 622, 590, and 540.5 U/ml, respectively. C2 sIL-2R was significantly decreased, as compared to pretreatment level followed by a plateau in subsequent cycles. The receiver operating characteristic curve determined 1123.5 U/ml as a cut-off value to predict progression-free survival (PFS) at 24 months (Fig. S1b, area under the curve, 0.769; 95% confidence interval [CI] = 0.723–0.816). Applying this cut-off value to sIL-2R pretreatment and C2 level, patients were classified into FINE subtype with pretreatment low level (*N* = 379), RES subtype with pretreatment high and C2 low level (*N* = 148), and RET subtype with pretreatment high and C2 high level (*N* = 72). The serial sIL-2R measurement was displayed in Fig. S1c. Compared with FINE subtype, RET subtype showed high-risk clinical features, and were significantly associated with increased non-germinal center B-cell (non-GCB) and BCL2/MYC double expressor (DE) (Table S1). Upon rituximab, cyclophosphamide, doxorubicin, vincristine, and prednisone treatment, significant difference was observed among three subtypes in terms of treatment response and PFS at 12/24 months (Table S1).

In both training cohort (Fig. 1a) and validation cohort (Fig. 1b), three subtypes differed significantly in PFS and overall survival (OS). In univariate analysis, dynamic change of sIL-2R, revised international prognostic index (R-IPI), DE, and non-GCB were related to inferior PFS and OS (Fig. S2a). Moreover, in multivariate analysis, dynamic change of sIL-2R was an independent adverse prognostic factor for PFS (hazard ratio [HR] = 2.239, 95%CI = 1.760–2.849) and OS (HR = 2.758, 95%CI = 2.032–3.744) (Fig. S2b, Table S2). We further assessed the ability of sIL-2R dynamic change to predict outcomes according to R-IPI. In R-IPI “very good” group (R-IPI = 0), patients achieved over 90% for 5-year OS rate and there was no significant difference on PFS and OS among sIL-2R subtypes (Fig. S3a). In R-IPI “good” group (R-IPI = 1–2) (Fig. S3b) and “poor” group (R-IPI = 3–5) (Fig. S3c), dynamic change of sIL-2R retained a risk-discriminatory ability for PFS and OS. Together, dynamic change of sIL-2R is a simple and easy detection method in peripheral blood to predict long-term survival.

**Fig. 1:**
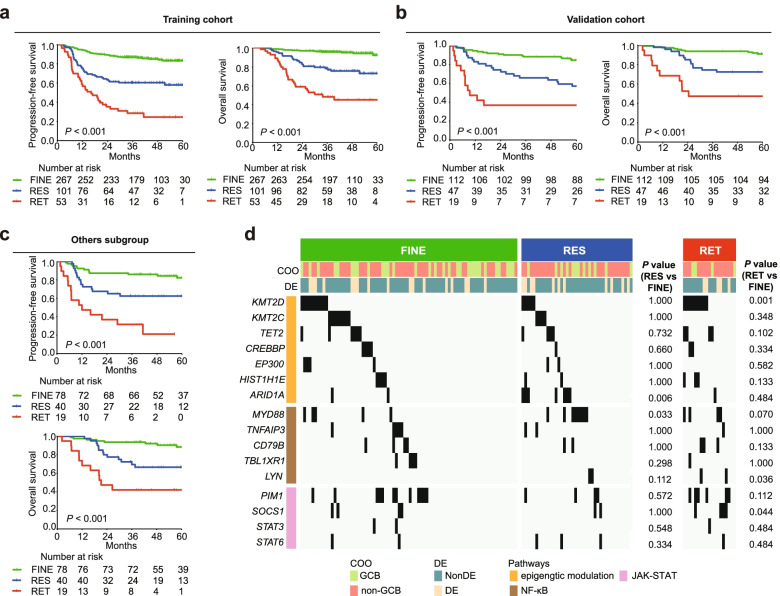
Prognostic significance and genetic features of sIL-2R subtypes. **a**, **b** Kaplan-Meier curves of progression-free survival (PFS) (upper panel) and overall survival (OS) (lower panel) for training cohort (**a**) and validation cohort (**b**), according to sIL-2R dynamic change. **c** Kaplan-Meier curves of PFS (upper panel) and OS (lower panel) according to sIL-2R dynamic change in “others” group. **d** Gene mutation profile in “others” subgroup according to sIL-2R subtypes. sIL-2R subtypes were indicated at the top and gene signatures were indicated at the left. *P* value was calculated between certain subtype and FINE subtype

Recently, genetic and lymphoma microenvironment (LME) subtypes of DLBCL demonstrated prognostic significance and therapeutic implications [8]. Whole exome and genome sequencing were screened in 223 patients, including 124 FINE, 70 RES and 29 RET subtypes. Eighty-six (38.6%) patients were genetically classified, while 137 patients were classified as “others” subgroup [9]. No significant difference of genetic subtypes was observed (Fig. S4a). Of note, dynamic change of sIL-2R remained prognostically significant for PFS and OS in “others” subgroup (Fig. 1c). Compared with FINE subtype, RES subtype had a higher mutation frequency in *ARID1A* and *MYD88*, and RET subtype had a higher mutation frequency in *KMT2D*, *LYN*, and *SOCS1* (Fig. 1d).

RNA sequencing was screened in 227 patients, including 126 FINE, 69 RES and 31 RET subtypes. With similar pattern of pathway alterations in the RES and RET subtypes (Table S3 and S4), we compared FINE subtype with combination of RES and RET subtypes. RES and RET subtypes showed significant enrichment in multiple oncogenic (apoptosis, ERK, JAK-STAT, MAPK, NF-κB) (Fig. 2a) and immune-associated pathways (Fig. 2b). The immunomodulatory effects of IL-2 are well-established pleiotropic on both effector T cells and Treg cells [10]. Compared with FINE subtype, as revealed by TIP method, RES subtype exhibited significantly increased recruiting activity of CD8 + T, T-helper 1, and natural killer cells (Fig. 2c), while RET subtype displayed increased recruiting activity of CD4 + T and Treg cells (Fig. 2d). Immune escape during cancer immunoediting compromised of activated immune regulatory cells and immune checkpoints [11]. As revealed by RNA sequencing, IL-2R was significantly associated with PD-1, CD47, CTLA-4, TIM-3, IL-10, LAG-3, PD-L1, TIGIT, and VISTA (Fig. S4b-c). JAK-STAT also showed correlation with inhibitory receptors, followed by NF-κB and ERK pathways (Fig. S4b). Moreover, protein-protein interaction analysis showed IL-10, PD-1, CTLA-4, and LAG-3 were associated with IL-2R and main proteins involved in JAK-STAT pathway (Fig. 2e). All 227 patients were also categorized by LME, including 57 depleted-, 32 GC-like, 67 inflammatory-, and 71 mesenchymal-LMEs [12]. RET subtype harbored significantly more inflammatory-LME than FINE subtype (Fig. 2f).

**Fig. 2:**
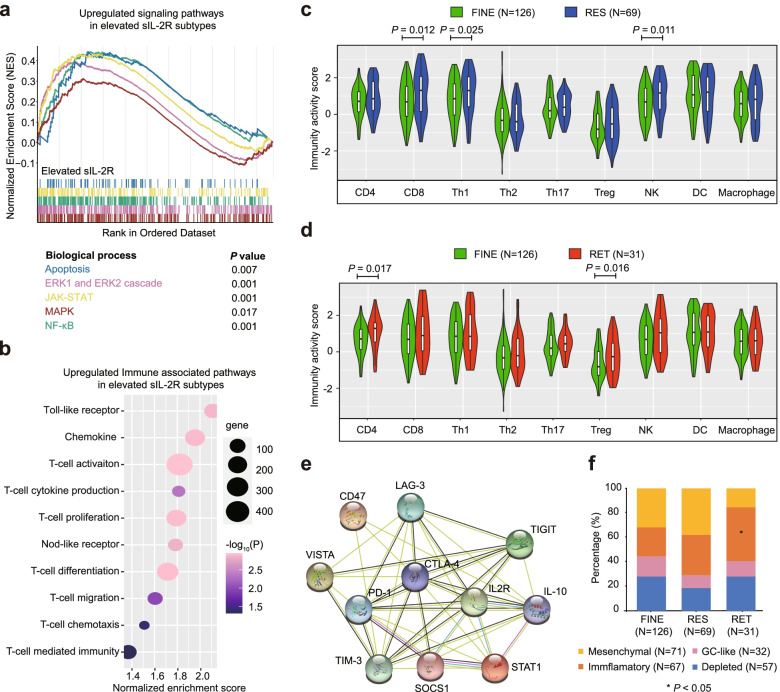
Lymphoma microenvironment of sIL-2R subtypes. **a** Gene Set Enrichment Analysis (GSEA) revealed oncogenic signaling pathway alterations in RES and RET subtypes compared with FINE subtype. **b** GSEA revealed immune-associated pathway alterations in RES and RET subtypes compared with FINE subtype. **c** Recruiting activity scores of immune cells between RES and FINE subtypes. Represented as mean ± SD. **d** Recruiting activity scores of immune cells between RET and FINE subtypes. Represented as mean ± SD. **e** Protein-protein interaction between IL-2R, multiple inhibitory receptors, and main proteins involved in JAK-STAT pathway. Green, black, pink and blue lines indicated interactions text mining, co-expression, experimentally-determined and from curated database, respectively. **f** The prevalence of lymphoma microenvironment (LME) classifications among sIL-2R subtypes. *P* value of specific LME was calculated between certain subtype and FINE subtype. * *P* < 0.05

To our knowledge, this is the first study to evaluate dynamic change of sIL-2R on prognostic significance and tumor microenvironment in DLBCL. With better understanding of sIL-2R biology profile, more clinical trials targeting sIL-2R vulnerability with surveillance of sIL-2R are warranted.

## Supplementary Information

**Additional file 1: Supplementary Methods. Figure S1.** Dynamic change of sIL-2R in DLBCL. **Figure S2.** Univariate and multivariate risk models in DLBCL according to sIL-2R dynamic change. **Figure S3.** Survival analysis in DLBCL according to sIL-2R dynamic change in patients risked by R-IPI. **Figure S4.** Genetic and lymphoma microenvironment features of sIL-2R subtypes. **Table S1.** Clinical and pathological characteristics of DLBCL patients. **Table S2.** Multivariate analysis and C-index for progression-free survival (PFS) and overall survival (OS) in DLBCL. **Table S3.** Pathway alterations in RES subtype. **Table S4.** Pathway alterations in RET subtype.

## Data Availability

Genomic and gene expression data have been deposited on National Omics Data Encyclopedia (NODE, https://www.biosino.org/node/ Project ID: OEP001143). All data are available without any restrictions. Correspondence and requests for materials should be addressed to W.-L.Z.

## Abbreviations

C2: Cycle 2
CI: Confidence interval
DE: Double expression
DLBCL: Diffuse large B-cell lymphoma
ERK: Extracellular signal-regulated kinase
HR: Hazard ratio
JAK: Janus kinase
LME: Lymphoma microenvironment
NF: Nuclear factor
non-GCB: Non-germinal center B-cell
OS: Overall survival
PFS: Progression-free survival
R-IPI: Revised International Prognostic Index
sIL-2R: Soluble interleukin-2 receptor
TIP: Tracking Tumor Immunophenotype
Treg: regulatory T
