# Supplementary material for: Dynamic change of soluble interleukin-2 receptor distinguished molecular heterogeneity and microenvironment alterations in diffuse large B-cell lymphoma
Source: Biomark Res. 2022 Jul 25;10:51. doi: 10.1186/s40364-022-00401-4 (PMC9316360; doi:10.1186/s40364-022-00401-4)
Supplement: Supplementary file 1 — Additional file 1: Supplementary Methods. Figure S1. Dynamic change of sIL-2R in DLBCL. Figure S2. Univariate and multivariate risk models in DLBCL according to sIL-2R dynamic change. Figure S3. Survival analysis in DLBCL according to sIL-2R dynamic change in patients risked by R-IPI. Figure S4. Genetic and lymphoma microenvironment features of sIL-2R subtypes. Table S1. Clinical and pathological characteristics of DLBCL patients. Table S2. Multivariate analysis and C-index for progression-free survival (PFS) and overall survival (OS) in DLBCL. Table S3. Pathway alterations in RES subtype. Table S4. Pathway alterations in RET subtype. [file 40364_2022_401_MOESM1_ESM.docx]

**Supplementary Materials for**

**Dynamic change of soluble interleukin-2 receptor distinguished molecular heterogeneity and microenvironment alterations in diffuse large B-cell lymphoma**

**Yu-Jia Huo^1†^, Peng-Peng Xu^1†^, Li Wang^1,2^, Hui-Juan Zhong^1^, Di Fu^1^, Qing Shi^1^, Shu Cheng^1^, Shuo Wang^1^, Mu-Chen Zhang^1^, Wei-Li Zhao^1,2*^**

**^†^** Contributed equally

^1^ Shanghai Institute of Hematology, State Key Laboratory of Medical Genomics, National Research Center for Translational Medicine at Shanghai, Ruijin Hospital Affiliated to Shanghai Jiao Tong University School of Medicine, Shanghai, China;

^2^ State Key Laboratory of Microbial Metabolism, School of Life Sciences and Biotechnology, Shanghai Jiao Tong University, Shanghai, China.

* Correspondence to: Wei-Li Zhao, Shanghai Institute of Hematology, State Key Laboratory of Medical Genomics, National Research Center for Translational Medicine at Shanghai, Ruijin Hospital Affiliated to Shanghai Jiao Tong University School of Medicine, 197 Rui Jin Er Road, Shanghai, 200025, China. Telephone: 0086 64370045, Email: [zhao.weili@yahoo.com](mailto:zhao.weili@yahoo.com).

**This PDF file includes:**

Supplementary Methods

Supplementary Figures. S1 to S4

Supplementary Tables. S1 to S4

**Supplementary Methods**

**1. Patients**

A flow chart describing the cohort selection was described in Figure 1A. A total of 599 patients with de novo DLBCL had serial serum measurement of sIL-2R based on registry data from January 2005 to December 2020, including a training cohort of 421 patients received R-CHOP retrospectively in a single Institute between August 2012 and April 2018, and a validation cohort of 178 patients received R-CHOP50, R-CEOP70 or R-CEOP90 prospectively from a multi-center, randomized clinical trial (NCT01852435) of the Multi-center Hematology Oncology Programs Evaluation System (M-HOPES) in China.^1^ All patients were diagnosed according to World Health Organization (WHO) classification.^2^ IPI and R-IPI were calculated as previously described.^3,4^ Hans classification and BCL2/MYC double expression (DE) was estimated as previously report.^5,6^ The study was approved by the Shanghai Ruijin Hospital Review Board with informed consent obtained in accordance with the Declaration of Helsinki.

**2. Cytokine assessment**

Serial serum specimens were collected pretreatment and before each cycle of the treatment. As for cytokines, sIL-2R were assessed by IMMUNITE 1000 chemiluminescence analyzer (Siemens) as previously reported.^7^ The lower limit of detection (LOD) and upper LOD for sIL-2R were 5 U/ml and 7500 U/ml, respectively.

**3. DNA sequencing**

Genomic DNA was extracted from frozen or formalin-fixed paraffin-embedded (FFPE) tumor samples using a QIAamp DNA Mini Kit (Qiagen, Duesseldorf, Germany) or a QIAamp DNA FFPE Tissue Kit (Qiagen), respectively, based on the manufacturer’s guidelines. Matched peripheral blood DNA was extracted using a QIAamp DNA Mini Kit (Qiagen, Duesseldorf, Germany). Whole exome sequencing (WES, n=157) and whole genome sequencing (WGS, n=66) were performed in 223 patients with frozen or qualified formalin-fixed paraffin-embedded tumor samples and were referred in our previous study.^8^ WES (n=25, divided into five groups) and WGS (n=17) were performed on 42 matched peripheral blood samples randomly selected to build a somatic mutation calling principle and to exclude germ-line polymorphisms.

For 66 patients, WGS was performed on frozen tumor tissue. Genomic DNA concentrations were measured with the Qubit (Thermo Fisher Scientific), and sheared to about 300bp fragments by Covaris DNA shearing system. After end-repaired and 3’-ends adenylated, Illumina PE adapters were ligated to DNA fragments to generate indexed library. Library was validated by Agilent 2100 Bioanalyzer and sequencing was performed on Illumina HiSeq platform with 150bp paired-end strategy in WuXi NextCODE, Shanghai. For 157 patients, WES was carried out on frozen tumor tissue (n=113), or on FFPE tumor tissue quality controlled by agarose gel electrophoresis (n=44). Exome regions were captured by a SeqCap EZ Human Exome kit (version 3.0) and sequencing was performed on HiSeq 4000 platform with 150bp paired-end strategy in Righton, Shanghai.

By Burrows-Wheeler Aligner (BWA) version 0.7.13-r1126, read pairs were aligned to RefSeq hg19 (downloaded from UCSC Genome Browser, URLs). Samtools version 1.3 was used to generate chromosomal coordinate-sorted bam files and to remove PCR duplications. The reads were then realigned around potential indel regions by Genome Analysis Toolkit (GATK) version 3.4 IndelRealigner with the recommended pipeline. Of each sample, the mean depth measured with WES/WGS was 120.25 × (range 50-200 ×), with an average 97.65% (range 82.64%-99.06%) of the target sequence being covered sufficiently deep for variant calling (≥ 10 × coverage).

GATK Haplotype Caller and GATK Unified Genotyper were applied to call SNVs and indels. Homemade pipeline was used to filter SNVs and indels detected by the above software, excluding: 1) mutations reported with low confidence; 2) germline mutations detected from control samples; 3) population-related variants reported in 1000 Genomes (dbSNP 137) as common SNPs and not included in COSMIC (the Catalogue of Somatic Mutations in Cancer) version v77. SNVs and indels were mapped to the genome location using the UCSC Genome Browser ([http://genome.ucsc.edu](http://genome.ucsc.edu/)). All the somatic functional mutations, including nonsynonymous SNVs, frameshift or in-frame indels, stopgain or stoploss and so on were obtained. Visual inspection was used to exclude potential false positive results.

Based on the WES and WGS results, we identified 55 recurrent and functional mutated genes of DLBCL.^8,9^ Sanger sequencing was used to confirm somatic mutations not observed in matched peripheral blood mononuclear cells. The tumor mutation burden was calculated as the total number of nonsynonymous SNVs, frameshift or in-frame indels, stopgain or stoploss mutations in above 55 DLBCL genes.

**4. RNA sequencing and bioinformatics analysis**

Total RNA was extracted from frozen tumor tissue by Trizol and RNeasy Mini Kit (Qiagen). RNA sequencing was performed in 227 patients as previously reported.^8^ RNA was purified using Ribo-Zero rRNA Removal Kits (Illumina). RNA concentration and integrity were verified using NanDrop and Agilent 2100 Bioanalyzer, respectively. Sequencing library was constructed using TruSeq RNA Samples Preparation Kit (Illumina). Qubit (Thermo Fisher Scientific) was used to quantify concentration of the resulting sequencing libraries, whereas the size distribution was analyzed using Agilent BioAnalyzer 2100 (Agilent). After library validation, clusters were generated by Illumina cBOT cluster generation system with HiSeq PE Cluster Kits (Illumina). Paired-end sequencing was performed using an Illumina HiSeq system following Illumina-provided protocols for 2×150 paired-end sequencing. Transcript counts table files were generated by the HTSeq using the GENCODE annotation database and processed with the BAM files generated by Hisat2.

Bioinformatic analyses were performed by r 3.6.1, using R package “sva” to remove batch effect. Limma (version 3.34.9) were used to normalize the raw reads and obtained differentially expressed genes (DEGs). Gene Set Enrichment Analysis (GSEA) was performed using the GSEA (v2.2.3, http://software.broadinstitute.org/gsea/downloads.jsp) with MSigDB-curated gene sets (c2.cp.kegg.v6.2.symbols.gmt) and (c5.all.v7.1.symbols.gmt).^10^ Tumor microenvironment was analyzed using Tracking Tumor Immunophenotype (TIP) (http://biocc.hrbmu.edu.cn/TIP/).^11^ Interaction of multiple proteins was analyzed using STRING in homo sapiens (https://cn.string-db.org/).

**5. Molecular classification**

For 223 patients with WES/WGS data, DLBCL genotypes were identified using the LymphGen probabilistic classification tool (R code version, https://doi.org/10.5281/zenodo.3700087) as described by Wright et al.^12^ Genetic aberrations including mutations, copy-number alterations, and fusions were analyzed and integrated. For 227 patients with RNA sequencing data, LME subtypes were classified according to methods described by Kotlov et al (R code version, https://github.com/bostongene/lme).^13^

**6. Statistical Analysis**

Fisher's exact tests were applied to compare non-ordinal categorical variables. Two-sided χ2 test were applied to compare continuous variables. Data was represented as mean ± SD. Correlation was assessed by Pearson’s correlation analysis. Concordance index (C-index, Harrell) was calculated to evaluate prognostic efficacy. Cut-off value was determined by the receiver operating characteristic curve with highest Youden index. Progression-free survival was measured from the date of diagnosis to the date when disease progression/relapse was recognized or the date of last follow-up. Overall survival was calculated from the date of diagnosis to the date of death or the date of last follow-up. Survival functions were estimated using the Kaplan-Meier method and compared by log-rank test. Univariate and multivariable hazard estimates were generated with Cox proportional hazards models. Covariates with *P* < 0.10 on univariate analysis were included in multivariate model. All statistical analysis was carried out using Statistical Package for the Social Sciences (SPSS) 25.0 software (SPSS Inc., Chicago, IL, USA) and R studio (version 3.6.1). *P* < 0.05 was considered statistically significant.

**
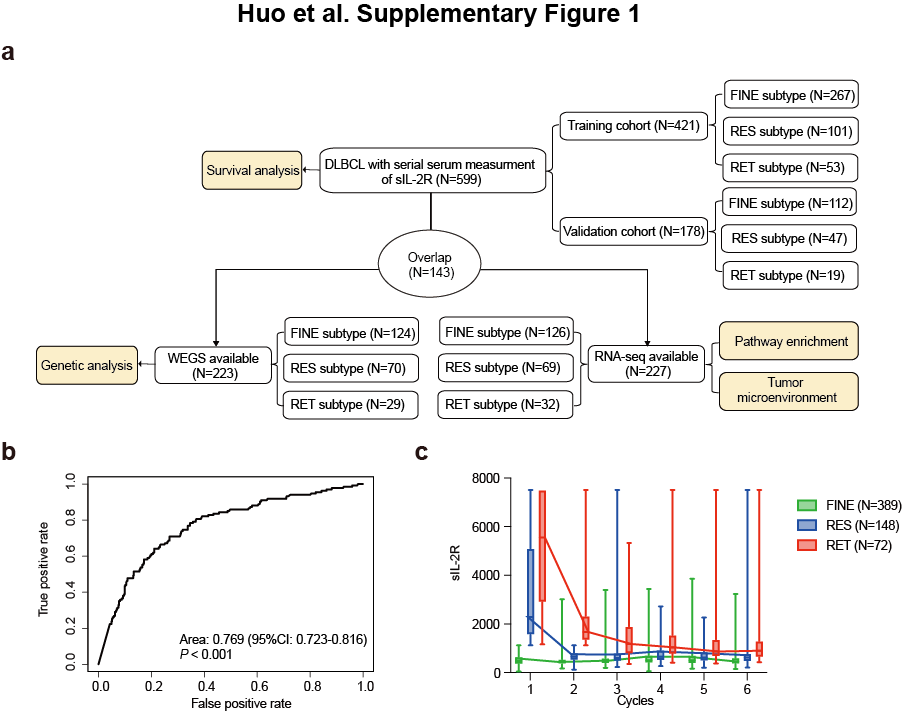
**

**Figure S1. Dynamic change of sIL-2R in DLBCL.**

(a) Flowchart of the patient selection. Upon R-CHOP treatment, a total of 599 patients with de novo DLBCL had serial serum measurement of sIL-2R, including a training cohort of 421 patients and a validation cohort of 178 patients. WES/WGS and RNA sequencing were performed in 223 and 227 patients, respectively. (b) The cut-off value of sIL-2R to predict progression-free survival at 24 months by the receiver operating characteristic curve. Area under the curve and 95% confidence interval were displayed. (c) The serial sIL-2R serum measurement pretreatment and before each cycle of treatment. Represented as mean ±SD.

**Figure S2. Univariate and multivariate risk models in DLBCL according to sIL-2R dynamic change**

(a) Univariate risk models for PFS (upper panel) and OS (lower panel) in DLBCL using four predictors (sIL-2R dynamic change, R-IPI, DE, and non-GCB). (b) Multivariate risk models for PFS (upper panel) and OS (lower panel) in DLBCL using predictors with *P* < 0.10 on univariate risk models. Hazard ratio was represented with 95% confidence interval (95% CI).

**Figure S3. Survival analysis in DLBCL according to sIL-2R dynamic change in patients risked by R-IPI**

Kaplan-Meier curves of progression-free survival (PFS) (upper panel) and overall survival (OS) (lower panel) for R-IPI “very good” group (R-IPI = 0) (a), “good” group (R-IPI = 1-2) (b), “poor” group (R-IPI = 3-5) (c), according to dynamic change of sIL-2R.

**Figure 4. Genetic and lymphoma microenvironment features of sIL-2R subtypes.**

(a) The prevalence of DLBCL genotypes defined by the LymphGen probabilistic classification tool among sIL-2R subtypes. (b) Correlation between inhibitory receptors and transcripts of IL-2R (upper panel), as well as score of signaling pathways (lower panel) using Pearson’s correlation analysis. (c) Significant difference in gene expression of inhibitory receptors between IL-2R low and IL-2R high subgroups divided by median transcripts of IL-2R.

**Table S1. Clinical and pathological characteristics of DLBCL patients.**

|  | **Overall**  **(n=599)**  **n (%)** | **FINE**  **(n=379)**  **n (%)** | **RES**  **(n=148)**  **n (%)** | **RET**  **(n=72)**  **n (%)** | ***P* value** |
| --- | --- | --- | --- | --- | --- |
| Age>60 | 249 (42) | 148 (39) | 61 (46) | 34 (47) | 0.216 |
| Gender, male | 319 (53) | 195 (51) | 84 (57) | 40 (56) | 0.514 |
| ECOG, 2-4 | 58 (10) | 10 (3) | 24 (16) | 17 (24) | <0.001 |
| Ann Arbor stage, III-IV | 259 (43) | 96 (25) | 107 (72) | 60 (83) | <0.001 |
| ≥2 extranodal sites | 222 (22) | 65 (17) | 60 (41) | 35 (49) | <0.001 |
| Elevated LDH | 273 (46) | 100 (26) | 112 (76) | 60 (83) | <0.001 |
| R-IPI |  |  |  |  | <0.001 |
| 0 | 147 (25) | 138 (36) | 8 (5) | 1 (1) |  |
| 1-2 | 271 (45) | 188 (50) | 61 (41) | 62 (31) |  |
| 3-5 | 181 (30) | 53 (14) | 79 (53) | 49 (68) |  |
| Non-GCB (n=536) | 332 (62) | 210 (58) | 96 (67) | 47 (72) | 0.030 |
| DE (n=526) | 109 (20) | 60 (18) | 32 (25) | 17 (30) | 0.034 |
| Treatment response |  |  |  |  | <0.001 |
| CR/PR | 526 (88) | 355 (94) | 129 (87) | 42 (58) |  |
| SD/PD | 73 (12) | 24 (6) | 19 (13) | 30 (42) |  |
| PFS12 | 86 (14) | 21 (6) | 33 (16) | 32 (44) | <0.001 |
| PFS24 | 134 (22) | 39 (10) | 49 (33) | 46 (64) | <0.001 |

Abbreviations: DE, double expression; ECOG, Eastern Cooperative Oncology Group; GCB, germinal center B-cell; LDH, lactate dehydrogenase; PFS12, progression-free survival at 12 months; PFS24, progression-free survival at 24 months; R-IPI: revised international prognostic index**Table S2. Multivariate analysis and C-index for progression-free survival (PFS) and overall survival (OS) in DLBCL.**

| **Characteristics** | **PFS** | | | **OS** | | |
| --- | --- | --- | --- | --- | --- | --- |
|  | **Multivariate analysis** | | **C-index** | **Multivariate analysis** | | **C-index** |
|  | P value | HR (95%CI) |  | P value | HR (95%CI) |  |
| R-IPI | 0.005 | 1.495 (1.133,1.972) | 0.679 | 0.34 | 1.748 (1.196, 2.556) | 0.710 |
| Non-GCB | 0.781 | 1.053 (0.731, 1.518) | 0.540 | / | / | 0.543 |
| DE | 0.033 | 1.493 (1.034, 2.156) | 0.559 | 0.12 | 1.579 (1.000, 2.492) | 0.566 |
| sIL-2R | <0.001 | 2.239 (1.760, 2.849) | 0.706 | <0.001 | 2.758 (1.032, 3.744) | 0.750 |

Abbreviations: DE, double expression; GCB, germinal center B-cell; R-IPI: revised international prognostic index

**Table S3. Pathway alterations in RES subtype.**

| **Description** | **Size** | | **NES** | ***P* value** |
| --- | --- | --- | --- | --- |
| KEGG_CYTOKINE_CYTOKINE_RECEPTOR_INTERACTION | | 262 | 1.697 | 0.001 |
| KEGG_CHEMOKINE_SIGNALING_PATHWAY | | 188 | 1.677 | 0.001 |
| KEGG_TOLL_LIKE_RECEPTOR_SIGNALING_PATHWAY | | 102 | 1.823 | 0.001 |
| KEGG_TYPE_I_DIABETES_MELLITUS | | 43 | 2.023 | 0.001 |
| KEGG_INTESTINAL_IMMUNE_NETWORK_FOR_IGA_PRODUCTION | | 48 | 1.918 | 0.001 |
| KEGG_SYSTEMIC_LUPUS_ERYTHEMATOSUS | | 55 | 2.095 | 0.001 |
| KEGG_AUTOIMMUNE_THYROID_DISEASE | | 52 | 2.019 | 0.001 |
| KEGG_NOD_LIKE_RECEPTOR_SIGNALING_PATHWAY | | 62 | 1.807 | 0.001 |
| KEGG_LEISHMANIA_INFECTION | | 72 | 2.228 | 0.001 |
| KEGG_ALLOGRAFT_REJECTION | | 37 | 2.120 | 0.001 |
| KEGG_DRUG_METABOLISM_CYTOCHROME_P450 | | 72 | -2.19 | 0.002 |
| KEGG_BUTANOATE_METABOLISM | | 34 | -1.77 | 0.002 |
| KEGG_RETINOL_METABOLISM | | 64 | -2.22 | 0.002 |
| KEGG_METABOLISM_OF_XENOBIOTICS_BY_CYTOCHROME_P450 | | 70 | -2.23 | 0.002 |
| KEGG_STARCH_AND_SUCROSE_METABOLISM | | 50 | -1.76 | 0.002 |
| KEGG_ARRHYTHMOGENIC_RIGHT_VENTRICULAR_CARDIOMYOPATHY_ARVC | | 74 | -1.84 | 0.002 |
| KEGG_DILATED_CARDIOMYOPATHY | | 90 | -1.71 | 0.002 |
| KEGG_DRUG_METABOLISM_OTHER_ENZYMES | | 51 | -1.87 | 0.002 |
| KEGG_RIBOSOME | | 87 | -2.44 | 0.002 |
| KEGG_HYPERTROPHIC_CARDIOMYOPATHY_HCM | | 83 | -1.62 | 0.002 |
| KEGG_FRUCTOSE_AND_MANNOSE_METABOLISM | | 33 | -1.69 | 0.004 |
| KEGG_CARDIAC_MUSCLE_CONTRACTION | | 73 | -1.53 | 0.004 |
| KEGG_GRAFT_VERSUS_HOST_DISEASE | | 41 | 1.723 | 0.005 |
| KEGG_ASTHMA | | 30 | 1.710 | 0.005 |
| KEGG_HUNTINGTONS_DISEASE | | 172 | -1.40 | 0.005 |
| KEGG_SPLICEOSOME | | 125 | -1.51 | 0.007 |
| KEGG_LYSOSOME | | 121 | 1.449 | 0.010 |
| KEGG_VIRAL_MYOCARDITIS | | 70 | 1.613 | 0.010 |
| KEGG_REGULATION_OF_ACTIN_CYTOSKELETON | | 212 | -1.36 | 0.011 |
| KEGG_FOCAL_ADHESION | | 199 | -1.36 | 0.011 |
| KEGG_ASCORBATE_AND_ALDARATE_METABOLISM | | 25 | -1.64 | 0.013 |
| KEGG_AXON_GUIDANCE | | 129 | -1.38 | 0.014 |
| KEGG_RENIN_ANGIOTENSIN_SYSTEM | | 17 | -1.61 | 0.014 |
| KEGG_GLYCOSPHINGOLIPID_BIOSYNTHESIS_LACTO_  AND_NEOLACTO_SERIES | | 26 | -1.61 | 0.015 |
| KEGG_STEROID_HORMONE_BIOSYNTHESIS | | 55 | -1.52 | 0.016 |
| KEGG_N_GLYCAN_BIOSYNTHESIS | | 46 | 1.537 | 0.017 |
| KEGG_NEUROACTIVE_LIGAND_RECEPTOR_INTERACTION | | 271 | -1.29 | 0.018 |
| KEGG_BIOSYNTHESIS_OF_UNSATURATED_FATTY_ACIDS | | 22 | 1.594 | 0.020 |
| KEGG_GLYCOLYSIS_GLUCONEOGENESIS | | 62 | -1.46 | 0.022 |
| KEGG_PENTOSE_AND_GLUCURONATE_INTERCONVERSIONS | | 28 | -1.56 | 0.022 |
| KEGG_PENTOSE_PHOSPHATE_PATHWAY | | 27 | -1.52 | 0.024 |
| KEGG_JAK_STAT_SIGNALING_PATHWAY | | 153 | 1.346 | 0.026 |
| KEGG_PURINE_METABOLISM | | 157 | -1.31 | 0.026 |
| KEGG_HISTIDINE_METABOLISM | | 29 | -1.51 | 0.032 |
| KEGG_ANTIGEN_PROCESSING_AND_PRESENTATION | | 88 | 1.372 | 0.032 |
| KEGG_FATTY_ACID_METABOLISM | | 42 | -1.45 | 0.032 |
| KEGG_CALCIUM_SIGNALING_PATHWAY | | 177 | -1.29 | 0.033 |
| KEGG_CYTOSOLIC_DNA_SENSING_PATHWAY | | 54 | 1.437 | 0.034 |
| KEGG_PPAR_SIGNALING_PATHWAY | | 69 | -1.37 | 0.036 |
| KEGG_TIGHT_JUNCTION | | 130 | -1.32 | 0.038 |
| KEGG_ERBB_SIGNALING_PATHWAY | | 87 | -1.33 | 0.038 |
| KEGG_OXIDATIVE_PHOSPHORYLATION | | 116 | -1.30 | 0.038 |
| KEGG_COMPLEMENT_AND_COAGULATION_CASCADES | | 67 | 1.420 | 0.039 |
| KEGG_PROXIMAL_TUBULE_BICARBONATE_RECLAMATION | | 23 | -1.41 | 0.043 |
| KEGG_CELL_ADHESION_MOLECULES_CAMS | | 131 | 1.324 | 0.048 |

**Table S4. Pathway alterations in RET subtype.**

| **Description** | **Size** | **NES** | ***P* value** |
| --- | --- | --- | --- |
| KEGG_PATHWAYS_IN_CANCER | 325 | 1.554 | 0.001 |
| KEGG_CYTOKINE_CYTOKINE_RECEPTOR_INTERACTION | 262 | 2.118 | 0.001 |
| KEGG_CHEMOKINE_SIGNALING_PATHWAY | 188 | 2.029 | 0.001 |
| KEGG_JAK_STAT_SIGNALING_PATHWAY | 153 | 1.796 | 0.001 |
| KEGG_TOLL_LIKE_RECEPTOR_SIGNALING_PATHWAY | 102 | 2.130 | 0.001 |
| KEGG_FC_GAMMA_R_MEDIATED_PHAGOCYTOSIS | 96 | 1.969 | 0.001 |
| KEGG_APOPTOSIS | 87 | 1.735 | 0.001 |
| KEGG_LEISHMANIA_INFECTION | 72 | 2.011 | 0.001 |
| KEGG_SYSTEMIC_LUPUS_ERYTHEMATOSUS | 55 | 1.845 | 0.001 |
| KEGG_COMPLEMENT_AND_COAGULATION_CASCADES | 67 | 1.849 | 0.001 |
| KEGG_ETHER_LIPID_METABOLISM | 33 | 1.939 | 0.001 |
| KEGG_O_GLYCAN_BIOSYNTHESIS | 30 | 1.779 | 0.001 |
| KEGG_LYSOSOME | 121 | 1.631 | 0.002 |
| KEGG_GLYCEROPHOSPHOLIPID_METABOLISM | 77 | 1.707 | 0.002 |
| KEGG_TYPE_I_DIABETES_MELLITUS | 43 | 1.695 | 0.003 |
| KEGG_RIBOSOME | 87 | -3.21 | 0.003 |
| KEGG_ENDOCYTOSIS | 181 | 1.478 | 0.003 |
| KEGG_ECM_RECEPTOR_INTERACTION | 83 | 1.611 | 0.004 |
| KEGG_PARKINSONS_DISEASE | 112 | -1.96 | 0.004 |
| KEGG_OXIDATIVE_PHOSPHORYLATION | 116 | -2.34 | 0.004 |
| KEGG_ADHERENS_JUNCTION | 73 | 1.659 | 0.004 |
| KEGG_NOD_LIKE_RECEPTOR_SIGNALING_PATHWAY | 62 | 1.716 | 0.004 |
| KEGG_LINOLEIC_ACID_METABOLISM | 29 | 1.705 | 0.004 |
| KEGG_ALZHEIMERS_DISEASE | 157 | -1.61 | 0.004 |
| KEGG_ALPHA_LINOLENIC_ACID_METABOLISM | 19 | 1.835 | 0.004 |
| KEGG_HUNTINGTONS_DISEASE | 172 | -1.68 | 0.005 |
| KEGG_TGF_BETA_SIGNALING_PATHWAY | 85 | 1.625 | 0.005 |
| KEGG_SMALL_CELL_LUNG_CANCER | 84 | 1.650 | 0.005 |
| KEGG_ARACHIDONIC_ACID_METABOLISM | 57 | 1.639 | 0.007 |
| KEGG_TASTE_TRANSDUCTION | 51 | 1.639 | 0.007 |
| KEGG_SPLICEOSOME | 125 | -1.61 | 0.008 |
| KEGG_OLFACTORY_TRANSDUCTION | 375 | -1.52 | 0.008 |
| KEGG_PENTOSE_AND_GLUCURONATE_INTERCONVERSIONS | 28 | -1.63 | 0.008 |
| KEGG_RNA_POLYMERASE | 29 | -1.69 | 0.008 |
| KEGG_DRUG_METABOLISM_OTHER_ENZYMES | 51 | -1.59 | 0.009 |
| KEGG_MAPK_SIGNALING_PATHWAY | 267 | 1.386 | 0.009 |
| KEGG_CARDIAC_MUSCLE_CONTRACTION | 73 | -1.50 | 0.010 |
| KEGG_ALLOGRAFT_REJECTION | 37 | 1.581 | 0.010 |
| KEGG_NEUROACTIVE_LIGAND_RECEPTOR_INTERACTION | 271 | 1.390 | 0.010 |
| KEGG_SPHINGOLIPID_METABOLISM | 39 | 1.599 | 0.012 |
| KEGG_BASAL_CELL_CARCINOMA | 55 | 1.482 | 0.027 |
| KEGG_GLYCOSPHINGOLIPID_BIOSYNTHESIS_LACTO_AND_NEOLACTO_SERIES | 26 | 1.550 | 0.027 |
| KEGG_ALDOSTERONE_REGULATED_SODIUM_REABSORPTION | 42 | 1.500 | 0.028 |
| KEGG_REGULATION_OF_ACTIN_CYTOSKELETON | 212 | 1.334 | 0.028 |
| KEGG_PRION_DISEASES | 35 | 1.505 | 0.031 |
| KEGG_CIRCADIAN_RHYTHM_MAMMAL | 13 | 1.584 | 0.033 |
| KEGG_SELENOAMINO_ACID_METABOLISM | 25 | 1.539 | 0.034 |
| KEGG_INOSITOL_PHOSPHATE_METABOLISM | 54 | 1.457 | 0.034 |
| KEGG_ASCORBATE_AND_ALDARATE_METABOLISM | 25 | -1.51 | 0.035 |
| KEGG_BETA_ALANINE_METABOLISM | 22 | 1.514 | 0.038 |
| KEGG_VASCULAR_SMOOTH_MUSCLE_CONTRACTION | 114 | 1.357 | 0.039 |
| KEGG_PHOSPHATIDYLINOSITOL_SIGNALING_SYSTEM | 76 | 1.404 | 0.040 |
| KEGG_GRAFT_VERSUS_HOST_DISEASE | 41 | 1.453 | 0.042 |
| KEGG_GLYCOSAMINOGLYCAN_DEGRADATION | 21 | 1.520 | 0.044 |
| KEGG_PEROXISOME | 78 | 1.390 | 0.046 |
| KEGG_DNA_REPLICATION | 36 | -1.40 | 0.049 |

**References**

1. Xu PP, Fu D, Li JY, Hu JD, Wang X, Zhou JF, et al. Anthracycline dose optimisation in patients with diffuse large B-cell lymphoma: a multicentre, phase 3, randomised, controlled trial. *Lancet Haematol* 2019; **6**(6): e328-e37.

2. Swerdlow SH, Campo E, Pileri SA, Harris NL, Stein H, Siebert R, et al. The 2016 revision of the World Health Organization classification of lymphoid neoplasms. *Blood* 2016; **127**(20): 2375-90.

3. A predictive model for aggressive non-Hodgkin's lymphoma. *N Engl J Med* 1993; **329**(14): 987-94.

4. Sehn LH, Berry B, Chhanabhai M, Fitzgerald C, Gill K, Hoskins P, et al. The revised International Prognostic Index (R-IPI) is a better predictor of outcome than the standard IPI for patients with diffuse large B-cell lymphoma treated with R-CHOP. *Blood* 2007; **109**(5): 1857-61.

5. Hans CP, Weisenburger DD, Greiner TC, Gascoyne RD, Delabie J, Ott G, et al. Confirmation of the molecular classification of diffuse large B-cell lymphoma by immunohistochemistry using a tissue microarray. *Blood* 2004; **103**(1): 275-82.

6. Hu S, Xu-Monette ZY, Tzankov A, Green T, Wu L, Balasubramanyam A, et al. MYC/BCL2 protein coexpression contributes to the inferior survival of activated B-cell subtype of diffuse large B-cell lymphoma and demonstrates high-risk gene expression signatures: a report from The International DLBCL Rituximab-CHOP Consortium Program. *Blood* 2013; **121**(20): 4021-31; quiz 250.

7. Zhong H, Chen J, Cheng S, Chen S, Shen R, Shi Q, et al. Prognostic nomogram incorporating inflammatory cytokines for overall survival in patients with aggressive non-Hodgkin's lymphoma. *EBioMedicine* 2019; **41**: 167-74.

8. Shen R, Xu PP, Wang N, Yi HM, Dong L, Fu D, et al. Influence of oncogenic mutations and tumor microenvironment alterations on extranodal invasion in diffuse large B-cell lymphoma. *Clin Transl Med* 2020; **10**(7): e221.

9. Huo YJ, Xu PP, Fu D, Yi HM, Huang YH, Wang L, et al. Molecular heterogeneity of CD30+ diffuse large B-cell lymphoma with prognostic significance and therapeutic implication. *Blood Cancer J* 2022; **12**(3): 48.

10. Subramanian A, Tamayo P, Mootha VK, Mukherjee S, Ebert BL, Gillette MA, et al. Gene set enrichment analysis: a knowledge-based approach for interpreting genome-wide expression profiles. *Proc Natl Acad Sci U S A* 2005; **102**(43): 15545-50.

11. Xu L, Deng C, Pang B, Zhang X, Liu W, Liao G, et al. TIP: A Web Server for Resolving Tumor Immunophenotype Profiling. *Cancer Res* 2018; **78**(23): 6575-80.

12. Wright GW, Huang DW, Phelan JD, Coulibaly ZA, Roulland S, Young RM, et al. A Probabilistic Classification Tool for Genetic Subtypes of Diffuse Large B Cell Lymphoma with Therapeutic Implications. *Cancer Cell* 2020; **37**(4): 551-68.e14.

13. Kotlov N, Bagaev A, Revuelta MV, Phillip JM, Cacciapuoti MT, Antysheva Z, et al. Clinical and Biological Subtypes of B-cell Lymphoma Revealed by Microenvironmental Signatures. *Cancer Discov* 2021; **11**(6): 1468-89.
